# Towards a molecular taxonomic key of the Aurantioideae subfamily using chloroplastic SNP diagnostic markers of the main clades genotyped by competitive allele-specific PCR

**DOI:** 10.1186/s12863-016-0426-x

**Published:** 2016-08-18

**Authors:** Amel Oueslati, Frederique Ollitrault, Ghada Baraket, Amel Salhi-Hannachi, Luis Navarro, Patrick Ollitrault

**Affiliations:** 1 Laboratoire de Génétique Moléculaire, Immunologie et Biotechnologie LR99ES12, Faculté des Sciences de Tunis (FST), Université de Tunis El Manar, Campus Universitaire, El Manar-Tunis, 2092 Tunisia; 2 Centro de Protección Vegetal y Biotecnología, Instituto Valenciano de Investigaciones Agrarias (IVIA), Moncada, 46113 Valencia Spain; 3 UMR Agap, CIRAD, Petit-Bourg, F-97170 Guadeloupe France

**Keywords:** Aurantioideae, Phylogeny, Clades, Chloroplastic SNP markers, KASPar™ genotyping

## Abstract

**Background:**

Chloroplast DNA is a primary source of molecular variations for phylogenetic analysis of photosynthetic eukaryotes. However, the sequencing and analysis of multiple chloroplastic regions is difficult to apply to large collections or large samples of natural populations. The objective of our work was to demonstrate that a molecular taxonomic key based on easy, scalable and low-cost genotyping method should be developed from a set of Single Nucleotide Polymorphisms (SNPs) diagnostic of well-established clades. It was applied to the Aurantioideae subfamily, the largest group of the Rutaceae family that includes the cultivated citrus species.

**Results:**

The publicly available nucleotide sequences of eight plastid genomic regions were compared for 79 accessions of the Aurantioideae subfamily to search for SNPs revealing taxonomic differentiation at the inter-tribe, inter-subtribe, inter-genus and interspecific levels. Diagnostic SNPs (DSNPs) were found for 46 of the 54 clade levels analysed. Forty DSNPs were selected to develop KASPar markers and their taxonomic value was tested by genotyping 108 accessions of the Aurantioideae subfamily. Twenty-seven markers diagnostic of 24 clades were validated and they displayed a very high rate of transferability in the Aurantioideae subfamily (only 1.2 % of missing data on average). The UPGMA from the validated markers produced a cladistic organisation that was highly coherent with the previous phylogenetic analysis based on the sequence data of the eight plasmid regions. In particular, the monophyletic origin of the “true citrus” genera plus *Oxanthera* was validated. However, some clarification remains necessary regarding the organisation of the other wild species of the Citreae tribe.

**Conclusions:**

We validated the concept that with well-established clades, DSNPs can be selected and efficiently transformed into competitive allele-specific PCR markers (KASPar method) allowing cost-effective highly efficient cladistic analysis in large collections at subfamily level. The robustness of this genotyping method is an additional decisive advantage for network collaborative research. The availability of WGS data for the main “true citrus” species should soon make it possible to develop a set of DSNP markers allowing very fine resolution of this very important horticultural group.

**Electronic supplementary material:**

The online version of this article (doi:10.1186/s12863-016-0426-x) contains supplementary material, which is available to authorized users.

## Background

Chloroplast DNA is a primary source of molecular variations for phylogenetic analysis of photosynthetic eukaryotes. The chloroplast genome has a simple and stable genetic structure, it is haploid, there are no (or very rare) recombination, it is generally uniparentally transmitted. It is known to be highly conserved in both gene order and gene content [1] with substitution rate much lower than nuclear DNA [2]. Therefore, universal primers can be used to amplify targeted sequences for molecular diversity analysis. Several fragments of coding regions, introns, and intergenic spacers, including atpB, atpB-rbcL, matK, ndhF, rbcL, rpl16, rps4-trnS, rps16, trnH-psbA, trnL-F, trnS-G, etc., have been used for phylogenetic reconstructions at various taxonomic levels [3–8]. Some of these regions such as matK, rbcL, and trnH-psbA have been relied upon heavily for development of candidate markers for plant DNA barcoding [9]. The matK gene is one of the most used sequence so far, because it is useful for identification at family, genus, and even species levels and trnH-psbA is the most variable region in the chloroplast genome across a wide range of groups [9]. Different kinds of molecular markers of chloroplast polymorphisms have been developed such as Restriction Fragment Length Polymorphisms (RFLPs), Cleaved Amplified Polymorphism Sequence (CAPS), Single Sequence Repeats (SSRs). More recently complete chloroplast genome sequences and whole genome resequencing data allowed very fine phylogenetic analysis [10]. Targeted or whole genome sequencing data offer the opportunity to identify polymorphisms diagnostic of well-defined clades. Our hypothesis is that the high level of conservation of the chloroplast sequence and the identification of clade-diagnostic Single Nucleotide Polymorphisms (SNPs) should allow developing molecular taxonomic keys at subfamily or family level, based on an easy, scalable and low-cost method of SNP marker genotyping. These diagnostic set of markers should then be applied in large germplasm collections. In this work we tested this hypothesis within the Aurantioideae subfamily (Rutaceae).

The Rutaceae family comprises seven subfamilies [11], of which the Aurantioideae is the largest group [12]. According to the Swingle and Reece classification [13] largely adopted by the citrus research community, the Aurantioideae subfamily comprises 33 genera and 210 species native to Africa, Australia, North and South America and Asia. They grow in varied climates from equatorial, hot-humid to cool maritime conditions. It is subdivided into two tribes: the Clauseneae with five genera and the Citreae with 28 genera. Swingle and Reece [13] believed that the Clauseneae tribe contained the more primitive genera of the subfamily. According to their classification, the Clauseneae tribe included three subtribes (Micromelinae, Clauseninae and Merrilliinae) and the Citreae tribe also includes three subtribes (Triphasilinae, Balsamocitrinae and Citrinae). The Citrinae is the most important subtribe of Citreae and comprise three groups: the “primitive citrus fruit trees”, the “near citrus fruit trees” and the “true citrus fruit trees”. The third group is formed by the *Citrus* genus and its closely related genera: *Fortunella* Swingle, *Microcitrus* Swingle, *Eremocitrus* Swingle, *Clymenia* Swingle and *Poncirus Raf* [13] [14]. Within the edible *Citrus*, molecular studies [15–23] identified four basic species: *C. medica* L. (citrons), *C. maxima* (Burm.) Osbeck (pummelos), *C. reticulata* Blanco (mandarins) and *C. micrantha* Wester (papeda). All other cultivated species, such as *C. sinensis* (sweet oranges), *C. aurantium* (sour oranges), *C. paradisi* (grapefruits), *C. lemon* (lemons) and *C. aurantifolia* (limes) result from these four ancestral taxa by reticulate evolution.

However, tribal and subtribal classifications of the Aurantioideae subfamily have barely been debated. Tanaka [24] grouped the subfamily into eight tribes and eight subtribes including 28 genera, while Engler [11] grouped all members of the subfamily into a single tribe, Aurantieae, subdivided into two subtribes, the Hesperethusinae (16 genera) and the Citrinae (13 genera). More recently, Mabberley [25] fused *Eremocitrus*, *Fortunella*, and *Microcitrus* with *Citrus*, and suggested that *Poncirus* should be so treated as well. These three classifications, as well as Swingle and Reece’s [13], are based on morphological traits generally influenced by environments and require strong human expertize for adequate classification inferences. In recent decades several molecular studies have been carried out to clarify the phylogenetic relationships within the Aurantioideae subfamily. The chloroplast genome of angiosperms is characterised by its evolutionary conservatism, the relative abundance of plant tissue, uniparental inheritance and small size (135 to 160 kb) [26]. Chloroplastic DNA (cpDNA) can avoid the problems of the complicated phylogenetic relationships within the Aurantioideae subfamily, and especially between *Citrus* and its close relatives, because of hybridization, apomixis and polyploidy [16]. Preliminary studies on the Aurantioideae subfamily using chloroplast DNA sequence data were based on two plastid genes, (the *atpB*-*rbcL* intergenic spacer and the *rps16* intron) analysed on 15 of the 33 genera from the Aurantioideae subfamily [27]. The polymorphisms provided were not informative enough for resolution or sufficient support for phylogenetic inferences. Araùjo et al. [28] published a phylogenetic study on the tribe Citreae, based on partial sequences from the *trnL*-*F* region and some morphological characters. Morton et al. [29] used the *rps16* and *trnL*-*trnF* introns from 24 genera. The results obtained by Samuel et al. [27], Araùjo et al. [28] and Morton et al. [29] were not fully congruent and did not have enough resolution to address tribal and subtribal delimitations.

Broader studies by Morton [30], and particularly by Bayer et al. [31], testing nine cpDNA gene regions confirmed the monophyly of the Aurantioideae subfamily and suggested none-monophyly for several subtribes. The study by Bayer et al. [31] resulted in a revision of the Swingle and Reece [13] classification and, still today, the more conclusive phylogenetic analysis of the whole Aurantioideae subfamily members.

Several studies tried to clarify the phylogeny within the “true citrus” group. For Bayer et al. [31] the circumscription of *Citrus genus* was broadened by including seven other closely related genera of the orange subfamily: *Clymenia*, *Fortunella*, *Poncirus*, *Microcitrus*, *Eremocitrus*, *Oxanthera* Montrouz., and *Feroniella* Swingle. The *Citrus* genus (as defined by Swingle and Reece [13]) was not monophyletic, with *C. medica* included in a clade with the Australian and New Caledonian genera. However, another study of the “true citrus fruit trees” group based on three cpDNA fragments (*trnL*-*trnF*, *psbH*-*petB* and *trnS*-*trnG*) confirmed its monophyly [32], but the group divided into six genera as previously proposed by Swingle and Reece [13]. A more definitive picture of the phylogeny of the true citrus group was recently provided by the analysis of complete chloroplast sequence polymorphisms [10] derived from whole genome resequencing data mapped on the sweet orange reference chloroplast genome [33]. It revealed three main clades: the first joining the citron with the Australian species, a second one associating the pummelos with *C. micrantha* and a third one the mandarins with *C. ichangensis*, a papeda species. *Poncirus* and *Fortunella* appeared as independent units.

Numerous studies analysed the maternal phylogeny within the *Citrus* genus using different tools. The oldest study was based on plastid Restriction Fragment Length Polymorphism (RFLPs) [26, 34, 35]. Other studies were based on chloroplast gene or spacer sequences such as *matK* sequences [36], *trnL*-*trnF* spacer [37, 38], *rbcL*-*ORF106*, *trnL*-*trnF* and *trnF*-*trnVr* region sequences [39] or *psbH*-*petB*, *trnL*-*trnF*, *rbcL* genes [40] and *trnS*-*trnG* cpDNA regions [41]. Chloroplastic Simple Sequence Repeats (CpSSRs) were also useful for differentiating the ancestral citrus taxa and for identifying the maternal phylogeny of secondary species [42–45]. Cleaved Amplified Polymorphic Sequence (CAPS) markers were also successfully developed [39, 46]. All these studies contributed to establishing the differentiation between the four ancestral taxa of the cultivated citrus (*C. reticulata*, *C. maxima*, *C. medica*, *C. micrantha*) and their contribution in the maternal phylogeny of the secondary species.

Two complete chloroplastic genome sequences have been published; the first for *C. sinensis* osbeck L (sweet oranges) [33] and more recently for *C. aurantifolia* (Omani lime cultivar) [47]. They revealed molecular polymorphisms (Insertion/ Deletion : Indels, Single Nucleotide Polymorphism: SNPs and SSRs) between the two species [47]. Based on whole genome resequencing data for 34 genotypes of the true citrus mapped on the chloroplast genome, Carbonell-Caballero et al. [10] identified 14.5 SNV/kb.

As mentioned before the objective of this work was to develop a molecular taxonomic key of the Aurantioideae subfamily based on an easy and low-cost SNP marker method. The competitive allele-specific PCR method allows efficient, scalable, easy and affordable SNP genotyping. It was therefore chosen for this study. The publicly available nucleotide sequence of eight plastid genomic regions (GeneBank; [31]) were compared for 79 accessions of the Aurantioideae subfamily to search for SNPs revealing taxonomic differentiation at inter-tribe, inter-subtribe, inter-genus and interspecific levels. KASPar markers were developed from the selected SNPs and used to genotype 108 accessions of the Aurantioideae subfamily to validate their taxonomic value, their transferability to the whole subfamily and to specify the maternal phylogeny of some *Citrus* species and cultivars.

## Methods

### Chloroplastic sequence selection

The sequences of eight chloroplastic regions used by Bayer et al. [31] for their phylogenetic study of the Aurantioideae subfamily, were obtained from the National Center of Biotechnology Information (NCBI reference, can be found in (Additional file 1): *atpB*-coding region, *rbcL*-*atpB* spacer, *rps16* spacer, *trnL*-*F* region, *rps4*-*trnT* spacer, *matK*-*5*’*trnK* spacer, *psbM*-*trnD*^*GUC*^ spacer and *trnG* intron. For each region, the sequences of seventy-nine accessions of the Aurantioideae subfamily were used. *Ruta graveolens* L. from the Rutaceae family was chosen as the outgroup. All genera of the Aurantioideae subfamily except *Limnocitrus* were sampled. The Clauseneae tribe was represented by three subtribes: Clauseninae (seven accessions), Merrilliinae (one accession) and Micromelinae (one accession). The Citreae tribe was represented by three subtribes: Triphasilinae (ten accessions), Balsamocitrinae (seven accessions) and Citrinae (fifty-three accessions). For the Citrinae subtribe we tried to select all known species of all genera (Table 1 and Table 2).

**Table 1:** Number of species/genus and plant accession/genus used for in silico SNP mining and KASPar analysis; classification according to Swingle and Reece [3]

Tribe	Subtribe		Genus	In Silico Mining		Kaspar experiment	
				NS	NA	NS	NA
Clauseneae	Micromelinae		*Micromelum*	1	1	0	0
	Clauseniae		*Clausena*	2	2	3	3
			*Glycosmis*	3	3	1	1
			*Murraya*	2	2	2	2
	Merrilliinae		*Merrillia*	1	1	1	1
Citreae	Triphasiinae		*Luvunga*	1	1	0	0
			*Merope*	1	1	0	0
			*Monanthocitrus*	1	1	0	0
			*Oxanthera*	2	2	1	1
			*Pamburus*	1	1	1	1
			*Paramignya*	2	2	1	1
			*Triphasia*	1	1	1	1
			*Wenzelia*	1	1	1	1
	Balsamocitrinae		*Aegle*	1	1	1	1
			*Aeglopsis*	1	1	1	1
			*Afraegle*	1	1	1	2
			*Balsamocitrus*	1	1	1	1
			*Feronia*	1	1	1	1
			*Feroniella*	1	1	1	1
			*Swinglea*	1	1	1	1
	Citrinae	Near Citrus Fruit	*Atalantia*	3	3	4	4
			*Citropsis*	2	2	4	4
		Primitive Citrus Fruit	*Hesperethusa*	1	1	1	1
			*Pleiospermium*	1	1	1	1
			*Burkillanthus*	1	1	0	0
			*Severinia*	1	1	2	2
		True Citrus fruit	*Citrus*	17	32	12	58
			*Clymenia*	1	1	1	1
			*Eremocitrus*	1	1	1	1
			*Fortunella*	3	3	6	6
			*Microcitrus*	6	6	8	8
			*Poncirus*	1	1	1	2

*NS* number of species, *NA* number of accession

**Table 2:** Species and number of plant accessions/species of the true citrus used for in silico SNP mining and KASPar analysis; classification according to Swingle and Reece [3]

Genus	Species	In silico SNP mining		KASPar analysis	
		NS	NA	NS	NA
Citrus	*C. maxima*	1	1	1	8
	*C. medica*	1	1	1	6
	*C. micrantha*	1	2	1	2
	*C. reticulata*	1	2	1	24
	*C.aurantifolia*	1	3	1	3
	*C. aurantium*	1	4	1	2
	*C. limon*	1	3	1	6
	*C. paradisis*	1	2	1	2
	*C. sinensis*	1	1	1	2
	*Others*	8	12	3	3
Clymenia	*Clymenia polyandra*	1	1	1	1
Eremocitrus	*E. glauca*	1	1	1	1
Fortunella	*F. hindsii*	0	0	1	1
	*F. crassifolia*	0	0	1	1
	*F. japonica*	1	1	1	1
	*F. margarita*	1	1	1	1
	*F. obavata*	0	0	1	1
	*F. polyandra*	1	1	1	1
Microcitrus	*M. australis*	1	1	1	1
	*M. australisica*	1	1	1	1
	*M. inodora*	1	1	1	1
	*M. garrowayae*	1	1	1	1
	*M. papuana*	1	1	1	1
	*M. virgata*	0	0	1	1
	*M. warburgiana*	1	1	1	1
	*M. australisica x C. mitis*	0	0	1	1
Poncirus	*P. trifoliata*	1	1	1	2

*NS* number of species, *NA* number of accession

### SNP identification and selection

Using BioEdit software [48], the sequences were aligned for each gene to the *C. sinensis* reference chloroplast genome sequence [33] available in [GenBank: NC008334]. The accessions were classed according to the consensus trees generated by Bayer et al. [31] to select SNPs between clades on different taxonomic levels. A SNP was considered as diagnostic of a clade when all accessions within the clade were identical and different from all the accessions outside the clade.

SNP loci were further selected for marker development using broad marker design criteria to allow utilization with several SNP chemistries. These criteria were: availability of DNA sequence 50 bp upstream and downstream from the SNP site and 15 bp around the target SNP with no additional molecular polymorphism.

### KASPar marker development and application

#### Plant material

One hundred and eight genotypes of the Aurantioideae subfamily, including five subtribes, namely the Balsamocitrinae, Citrinae, Triphasiinae (from the Citreae tribe) and Clauseninae and Merrilliinae (from the Clauseneae tribe) were sampled for the SNP markers selected. In this study, we adopted the Swingle and Reece subdivision [13]. The Clauseninae subtribe included three genera: *Clausena* (three accessions), *Glycosmis* (one accession) and *Murraya* (two accessions). The Merrilliinae subtribe was represented by the only genus *Merrillia* with one accession. The Balsamocitrinae subtribe was represented by seven genera: *Swinglea* (one accession), *Aegle* (one accession), *Afraegle* (two accessions), *Aeglopsis* (one accession), *Balsamocitrus* (one accession), *Feronia* (one accession), and *Feroniella* (one accession). The Triphasilinae subtribe included five genera: *Oxanthera*, *Pamburus*, *Paramignya*, *Triphasia*, and *Wenzelia*. One accession represented each genus. Within the Citrinae subtribe three groups were represented: the primitive citrus fruits, the near citrus fruits, and the true citrus fruits. The near citrus fruit group included two genera, *Citropsis* (four accessions) and *Atalantia* (four accessions), the primitive citrus fruit group included the genera *Severinia* (two accessions), *Pleiospermium* (one accession) and *Hesperethusa* (one accession), and the true citrus fruit group was represented by six genera, namely *Fortunella* (six accessions), *Eremocitrus* (one accession), *Poncirus* (two accessions), *Clymenia* (one accession), *Microcitrus* (eight accessions) and *Citrus* (fifty-eight accessions) (Table 1, Table 2 and detailed in (Additional file 2).

#### DNA extraction

High molecular weight genomic DNA was extracted from leaf samples using the DNeasy Plant Mini Kit (Qiagen S.A.; Madrid, Spain) according to the manufacturer’s instructions.

#### KASPar™ Genotyping

The KASPar™ Genotyping System from LGC Genomics® is a competitive allele-specific dual FRET based assay for SNP genotyping. It uses two Fluorescent Resonance Energy Transfert (FRET) cassettes where fluorometric dye, HEX or FAM, is conjugated to primer but quenched via resonance energy transfer. Sample DNA is amplified with a thermal cycler using allele-specific primers, leading to the separation of fluorometric dye and quencher when the FRET cassette primer is hybridized with DNA [49]. The primers were designed by LGC Genomics® based on the SNP locus-flanking sequence (approximately 50 nucleotides either side of the SNP). Detailed information for all SNP markers can be found in Additional file 3. The fluorescence signals of PCR products were measured with Fluostar Omega (BMG) and genotype calling was done with KlusterCaller software (LGC Genomics).

### SNP marker data analysis

#### Theoretical Clade differentiation

The GST parameter [50] was used to estimate the efficiency of each developed marker to effectively differentiate the clade identified by Bayer et al. [31]. GST estimations were computed using Excel software considering two subpopulations: (1) the accessions theoretically included in the clade in question, (Ci) and (2) a theoretical population of all other accessions (C-i). The analysis was performed from the estimated allele frequency of each group considering the same population size to estimate the frequency of the whole population (Tot) frequency.

$$ GS{T}_{\mathrm{C}\mathrm{ladei}}=\frac{H{e}_{\mathrm{Tot}}-\frac{\left( He{}_{\mathrm{C}\mathrm{i}}+ He{}_{\mathrm{C}\hbox{-} \mathrm{i}}\right)}{2}}{H{e}_{\mathrm{Tot}}} $$

Where He is the genetic diversity within population (*He* = 1-Σ *pi*^2^, where *pi* is the frequency of a given allele in the considered population).

***Polymorphism Information Content*** (***PIC***) was estimated for each marker as *PIC* = 1-Σ (*Pi*) ^2^

***Clade analysis***: a cladistic analysis was performed from the KASPar SNP data. Unweighted pair group method with the arithmetic average (UPGMA) was performed with Mega version 6 software [51] from a matrix of Euclidian distances between each pair of accessions.

## Results and discussion

### Clade-specific SNP identification

**Fig. 1:**
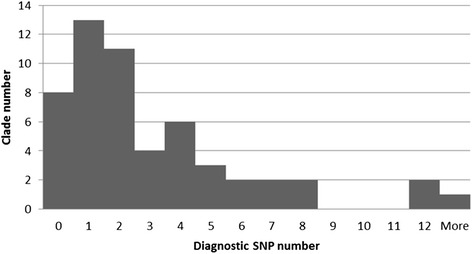
Distribution of the number of DSNPs for the 54 considered clades from Bayer et al. [21]

The available sequences of 79 Aurantioideae accessions for 8 chloroplastic genome regions were aligned and organized according to the Bayer et al. [31] classification (see Additional file 1 for the nomenclature of the Bayer Clades). They were also aligned to the reference chloroplastic genomes of sweet orange [33] in order to locate the selected SNPs on that reference genome. This allowed easy visual identification of SNPs totally differentiating the different clades from the rest of the accessions. Additional file 4 gives an example of such diagnostic SNP (DSNP) mining in the region *rps4*-*trnT* spacer for the Bayer clade Q corresponding to the true citrus of Swingle and Reece [13] plus the *Oxanthera* and *Feroniella* genera. In position 49301 of the reference chloroplastic genome a SNP (A/G) totally differentiated the accessions of clade Q (A) from all other Aurantioideae accessions (G). The alignments of the height analysed chloroplast regions can be found in the Additional file 5. Of the 54 clade levels analysed, 8 did not display any DSNPs. (one of them displaying a SNP shared by the clade GG1 and the species *C. aurantifolia* and *Hesperethusa crenulata*). The majority (28) displayed between one and 3 DSNPs, 15 clades displayed between 4 and 8 DSNPs and only three displayed 12 or more DSNPS. A total of 166 DSNPs was identified (Fig. 1 and Tables 3).

**Table 3:** Number of diagnostic SNPs of the Bayer’s Clade encountered from the in silico mining

Bayer's Clade	atpB-coding region		rbcL-atpB spacer		rps16 spacer		trnL-F region		rps4-trnT spacer		matK-5'trnK spacer		psbM-trnD^GUC^ spacer		trnG intron		Total	
	D	D'	D	D'	D	D'	D	D'	D	D'	D	D'	D	D'	D	D'	D	D'
A	0	0	0	0	1	0	1	2	0	0	4	2	3	1	3	0	12	5
B	0	0	2	0	0	0	0	0	1	0	0	0	0	0	0	0	3	0
C	0	0	1	0	1	0	0	2	0	0	0	0	0	0	2	0	4	2
D	1	0	3	0	0	0	0	0	0	0	0	0	0	0	0	0	4	0
E/G	0	0	1	0	0	0	0	1	3	0	1	0	0	0	0	0	5	1
F	2	0	1	1	1	0	0	0	1	0	0	0	3	0	0	1	8	2
H	0	0	0	0	0	0	0	0	0	0	0	0	1	0	1	0	2	0
I	0	0	0	0	0	0	0	0	0	0	0	0	0	0	0	0	0	0
J	0	0	0	1	0	0	0	0	0	0	0	0	1	0	0	0	1	1
K	2	0	0	0	2	3	0	0	0	0	1	0	0	0	3	0	8	3
L	2	0	2	0	0	0	0	0	0	0	0	0	0	0	0	0	4	0
L1	0	0	0	0	0	0	0	0	0	0	0	0	0	0	0	0	0	0
L2	3	0	0	0	1	0	0	0	0	0	0	0	0	0	0	0	4	0
L3	0	0	0	0	0	0	0	0	0	0	0	0	0	0	1	0	1	0
M	1	0	0	0	0	0	0	0	1	0	0	1	0	0	0	0	2	1
N	0	0	0	0	0	0	0	0	0	0	0	1	1	0	0	0	1	1
O	0	0	0	0	0	0	0	0	0	0	0	0	0	0	0	0	0	0
P	0	0	0	0	0	0	0	0	0	0	1	0	0	1	1	0	2	1
Q	0	2	1	1	0	0	0	0	1	0	1	0	0	0	4	0	7	3
R	0	0	0	0	0	0	0	0	0	0	0	0	1	0	0	0	1	0
S	0	0	1	0	0	0	0	0	0	0	0	0	0	0	0	0	1	0
T	0	0	0	0	0	0	0	0	0	0	0	0	0	0	0	0	0	0
U	0	0	0	0	1	0	0	0	0	0	1	0	1	0	1	0	4	0
V	0	0	0	0	0	0	0	0	1	0	1	0	2	0	2	1	6	1
W	0	0	0	0	0	0	0	0	0	0	1	0	0	0	0	0	1	0
X	0	0	1	0	0	0	0	0	0	0	0	0	0	0	1	1	2	1
Y	0	0	0	0	0	0	0	0	0	0	0	0	0	0	0	0	0	0
Z	0	0	0	0	1	0	0	0	0	0	0	0	1	0	1	0	3	0
AA	0	0	0	0	0	0	0	0	0	0	0	0	1	0	1	0	2	0
BB	0	0	0	0	0	0	0	0	0	0	0	0	0	0	1	0	1	0
CC	0	0	0	0	0	0	0	0	0	0	1	3	0	0	0	0	1	3
DD	0	0	0	0	1	1	1	0	0	0	0	0	0	0	0	0	2	1
EE	0	0	0	0	1	0	1	0	0	0	0	0	1	0	0	0	3	0
FF1	0	0	0	0	0	0	1	0	0	0	0	0	0	0	0	0	1	0
FF2	0	0	0	0	0	0	3	0	1	0	1	0	1	0	1	1	7	1
GG1	0	0	0	0	0	0	0	0	0	0	0	0	0	0	0	1	0	1
GG2	0	0	0	0	0	0	0	0	2	0	0	0	0	0	0	0	2	0
GG3	0	0	0	0	1	0	0	0	0	0	0	0	0	0	1	0	2	0
GG4	0	0	0	0	0	0	1	0	0	0	0	0	0	0	0	0	1	0
GG5	1	2	0	0	1	0	0	0	0	0	0	0	0	0	0	0	2	2
GG6	0	0	0	0	0	0	0	0	0	0	0	0	2	0	0	0	2	0
HH	1	0	1	0	0	0	0	0	0	0	0	0	2	0	1	0	5	0
II	0	0	0	0	1	1	1	1	0	0	0	0	0	0	0	0	2	2
JJ	0	0	0	0	0	0	1	0	0	0	0	0	0	1	0	0	1	1
KK	0	0	0	0	0	0	0	0	0	0	0	0	0	0	3	0	3	0
LL	0	0	0	0	1	0	0	0	1	0	0	0	1	0	1	0	4	0
MM	0	0	0	0	0	0	0	0	0	0	0	0	0	0	0	0	0	0
NN	0	0	0	0	0	0	0	0	0	0	0	0	0	0	0	0	0	0
OO	1	0	1	1	0	1	1	0	1	1	1	2	1	0	0	0	6	5
PP	3	0	2	3	2	0	2	0	0	0	1	0	3	0	1	0	14	3
QQ	0	0	2	0	2	1	0	0	0	0	1	0	0	0	0	0	5	1
RR	1	1	1	0	1	1	2	0	0	0	3	0	2	1	2	1	12	4
SS	0	0	0	0	0	0	0	0	0	0	1	0	0	0	0	0	1	0
TT	1	0	0	0	0	0	0	0	0	0	0	0	0	0	0	0	1	0
Total	19	5	20	7	19	8	15	6	13	1	20	9	28	4	32	6	166	46

D: marker totally diagnostic of the Clade; D’: markers shared by all accession of the Clade and very few accessions outside the Clade

For two of the clades with 12 or more DSNPs (*Poncirus trifoliata and Clymenia polyandra*), these high values could be partially explained by the fact that these taxa were represented by only one accession and that we therefore missed a major part of intra-taxa diversity. However, it also argues in favour of considerable differentiation of these genera within the “true citrus” clade. The third clade with a high DSNP value was the Glycosmis clade with accessions of three species, confirming the conclusion of Bayer et al. [31] and Samuel et al. [27] regarding the strong monophyletism of Glycosmis. The identification of 8 and 7 DSNPs for the clades F (*Merrillia* and *Murraya paniculata*), K (*Aegle*, *Aeglopsis*, *Afraegle* and *Balsamocitrus*) and Q (true citrus plus *Feroniella* and *Oxanthera*) validated the robustness of these clades and the monophyletic character of each one. For clade F, it confirmed the close relationship of *Merrillia* and *Murraya paniculata* proposed by Bayer et al. [31]. The K clade was defined by Bayer et al. as the true Balsamocitrinae clade corresponding to the bael-fruit group of Swingle and Reece [13]. The monophyletic origin of the Q clade including the sequences of the true Citrus [13], *Feroniella* and *Oxanthera*, is also clearly confirmed by these numerous DSNPs. Also with 7 DSNPs, *C. macroptera* appeared clearly differentiated from other papeda such as *C. micrantha* and *C. celebica*, even though they were joined in the DD clade validated with 2 DSNPs. The 6 DSNPs for the OO (*Oxanthera*) and V (*M. australasica*; *M. Garrawayi*) clades revealed substantial differentiation within the Australian/New Caledonian clade. With 5 DSNPs the *C. medica*/*C. indica* clade (QQ) was clearly validated. Only one DSNP was found for the R clade joining the *C. indica*/*C. med*ica clade and the Australian/New Caledonian one (T). It revealed the distended relationship between the two groups, even though the R clade was also inferred from Whole Genome Sequence (WGS) data [10]. The identification of 5 DSNPs for clade E and clade G strongly supported the monophyletic origins of *Bergera*/*Clausena*/*Micromelum*/*Glycosmis* on the one hand and all the other considered genera on the other hand. Also with 5 DSNPs *C. ichangensis* appeared clearly differentiated from the other papeda. With 4 DSNPs the monophyletic origins of clade C (*Clausena* species), D (*Bergera* and *Clausena*), L (*Merope*/*Monanthocitrus*/*Wenzelia*), and U (Australian citrus) were also clearly validated. The absence of DSNPs for several clades identified by Bayer et al. [31] corresponded to branching with a low maximum parsimony bootstrap in their consensus phylogenetic tree analysis and highlighted the weakness of these clades. It concerned the monophyletic origin of (i) clade I, *Triphasia trifolia*, and the strong clade *Merope*/*Monanthocitrus*/*Wenzelia*, (ii) clade O (*Feronia* and *Atalantia*) and (true citrus plus *0xanthera* and *Feroniella*). It therefore appears that the resolution of the Citreae tribe into sub-groups remains confused and data from whole chloroplast genome sequences will probably be necessary to definitively conclude on the organisation of this tribe. The monophyletic origin of the subsequent groups is also questioned: (iii) clade T: *Clymenia polyandra* and the Australian/New Caledonian clade, (iv) clade Y joining the clades BB (mandarins) and Z (pummelos and papedas). The other branching with no DSNPs concerned more recent differentiation within closely related accessions (L1; GG1; MM; NN).

Of the 166 DSNPs identified, 40 SNPs diagnostic of 33 clades (Additional file 3) were selected. For each clade the main selection criterion was the absence of additional polymorphisms (SNPs or Indels) in close vicinity (ideally in the 30 pb flanking the SNP each side; but at least in the 15 pb for clades when the previous condition was not encountered).

### KASPar analysis

Primers for the 40 selected SNPs were designed by LGC Genomics® based on the SNP locus-flanking sequence (approximately 50 nucleotides either side of the SNP (Additional file 3). Eleven of these markers provided an inconsistent or no signal and were not useful: CP_3707 (clade DD), CP_10192 (clade U), CP_32282 (clade N), CP_5927 (clade EE), CP_51032 (clade II), CP_32684 (clade Z), CP_57022 (clade A), CP_58121 (clade J), CP_51540 (clade JJ), CP_57256 (clade L), CP_3996 (clade Q). The CP_57686 marker, theoretically diagnostic of Ichang papeda, provided a good signal but was not polymorphic so it was discarded. Moreover, CP_57742, which was theoretically diagnostic of *Clymenia*, differentiated *Atalantia ceylanica* but not *Clymenia* from all the other accessions and was also discarded.

**Fig. 2:**
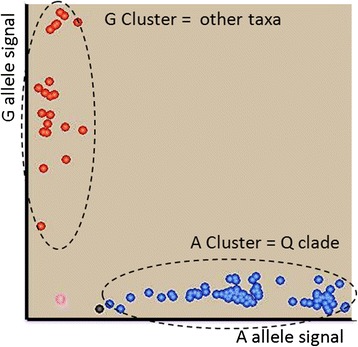
An example of KASPar analysis (marker CP_49301) distinguishing the accessions of the Bayer’s Clade Q. Q clade represent true Citrus of Swingle and Reece [3] plus Oxanthera. Red dots: SNP “A”, Blue dots: SNP “G”, Black dot: blank data, and Pink dot: missing data

A genetic analysis was performed with the 27 remaining markers diagnostic of 24 Bayer et al. [31] clades, for 108 accessions (Table 4). Figure 2 illustrates the clear differentiation obtained with the CP_49301 marker between the true citrus accessions of Swingle and Reece, plus *Oxanthera* and *Clymenia*, and all the other genera. The rate of missing data for these markers was very low, ranging from 0 to 2.8 % with an average of 1.2 %. This situation is very different from the one encountered for SNP KASPar nuclear markers mined from a panel of *Citrus* accessions where the frequency of missing data was higher for the citrus relatives and increased with taxonomic distances within the Aurantioideae subfamily [20]. This showed that selection based on the absence of additional polymorphism in close vicinity to the selected SNPs was efficient. This very good transferability also resulted from the much lower rate of chloroplast sequence evolution compared to nuclear evolution. The PIC varied from 0.02 to 0.5 in relation to the taxonomic level revealed by each marker, but also to our sample with an overrepresentation of the true citrus compared to the other genera.

**Table 4:** KASPar marker results

Marker	Position	Ref/Alt	Th. Clade	% MD	CDi	PIC
CP_58345	58345	G/A	QQ	2.8 %	1.00	0.11
CP_4296	4296	G/A	QQ	0.9 %	1.00	0.11
CP_32299	32294	G/T	R	4.6 %	1.00	0.28
CP_58401	58401	G/T	S	1.8 %	1.00	0.50
CP_56697	56697	T/C	D	0.0 %	1.00	0.07
CP_3807	3807	C/T	E	1.8 %	1.00	0.09
CP_58454	58454	C/T	G	1.8 %	1.00	0.09
CP_32526	32521	A/G	H	1.8 %	1.00	0.12
CP_3964	3964	C/A	Eremocitrus	1.8 %	1.00	0.02
CP_9149	9149	T/A	LL	0.9 %	1.00	0.02
CP_58062	58062	T/G	X	0.0 %	0.98	0.15
CP_10342	10343	C/A	AA	0.9 %	0.96	0.15
CP_51634	51634	C/A	FF	0.0 %	1.00	0.09
CP_3674	3674	C/G	V	0.0 %	1.00	0.05
CP_4243	4243	A/C	W	0.0 %	1.00	0.10
CP_57691	57691	T/C	RR	0.0 %	1.00	0.04
CP_4159	4159	A/C	CC	0.9 %	0.92	0.36
CP_57064	57064	G/A	F	2.8 %	1.00	0.04
CP_32463	32458	T/C	J	0.0 %	1.00	0.04
CP_9158	9158	T/C	K	0.9 %	1.00	0.09
CP_3829	3829	T/C	M	1.8 %	0.88	0.23
CP_49405	49405	A/G	M	2.8 %	0.98	0.24
CP_3747	3747	G/A	N	0.0 %	0.73	0.12
CP_51068	51068	A/C	OO	0.0 %	1.00	0.02
CP_3982	3982	C/T	P	1.8 %	0.96	0.12
CP_49301	49301	A/G	Q	0.9 %	1.00	0.41
CP_58553	58553	G/T	Q	0.0 %	1.00	0.41

*Th. Clade* clade differenciated by the SNP according to the in silico analysis, %*MD* percentage of missing data; *Cdi* Clade differenciation rate (Gst); *PIC* polymorphism information content

The GST analysis (the clade theoretically diagnosed by the SNP considered as one population and the rest of the sample as a second one) globally validated the cladistic diagnostic value of the selected markers with an average value of 0.98. Twenty of the markers totally fitted (GST = 1). For the marker of the CC clade (CP_4159) the Yuzu (*C. junos*) was different from the mandarins, while it was included in the Bayer et al. analysis [31]. Our results are in agreement with the results of Abkenar et al. [52] and Yamamoto et al. [39] who found the Yuzu differentiated from mandarins by Chloroplastic CAPS analysis. Yuzu was found to be closely associated with Ichang papeda in several studies [37, 39]. Therefore, except for Yuzu which still has a debated status, this marker appears as a perfect univocal diagnostic marker of the mandarin group. For the M clade the two selected markers displayed one outgroup accession with the same allele as clade M (*Clausena excavata* for CP_3829 and *Severinia disticha* for CP_49405). CP_58062 for clade X (*Fortunella* Sp *and C. halimii*) also displayed one accession outside the clade (*Atalantia ceylanica*), sharing the clade allele. For CP_10342 (clade AA; *Fortunella* sp.) the diagnostic allele was shared with two accessions of clade D (*Clausena anisata* and *Murraya koenigii*) in addition to the 6 *Fortunella* species analysed. For *M. koenigii* it confirmed the results of the SNP mining from the Bayer et al. [31] sequence alignments. For CP_3982 (clade P; *Limonia* and *Atalantia*) the diagnostic allele was shared with two accessions of clade N (*Hesperethusa* and *Pleiospermium*). For CP_3747 (clade N) displaying the lower GST value (0.73) one accession of the theoretical clade shared the out-clade allele while *Severinia buxifolia* shared the clade allele. The results of the last two markers confirmed the weakness of the resolution of the Citrinae clade into consistent subclades.

**Fig. 3:**
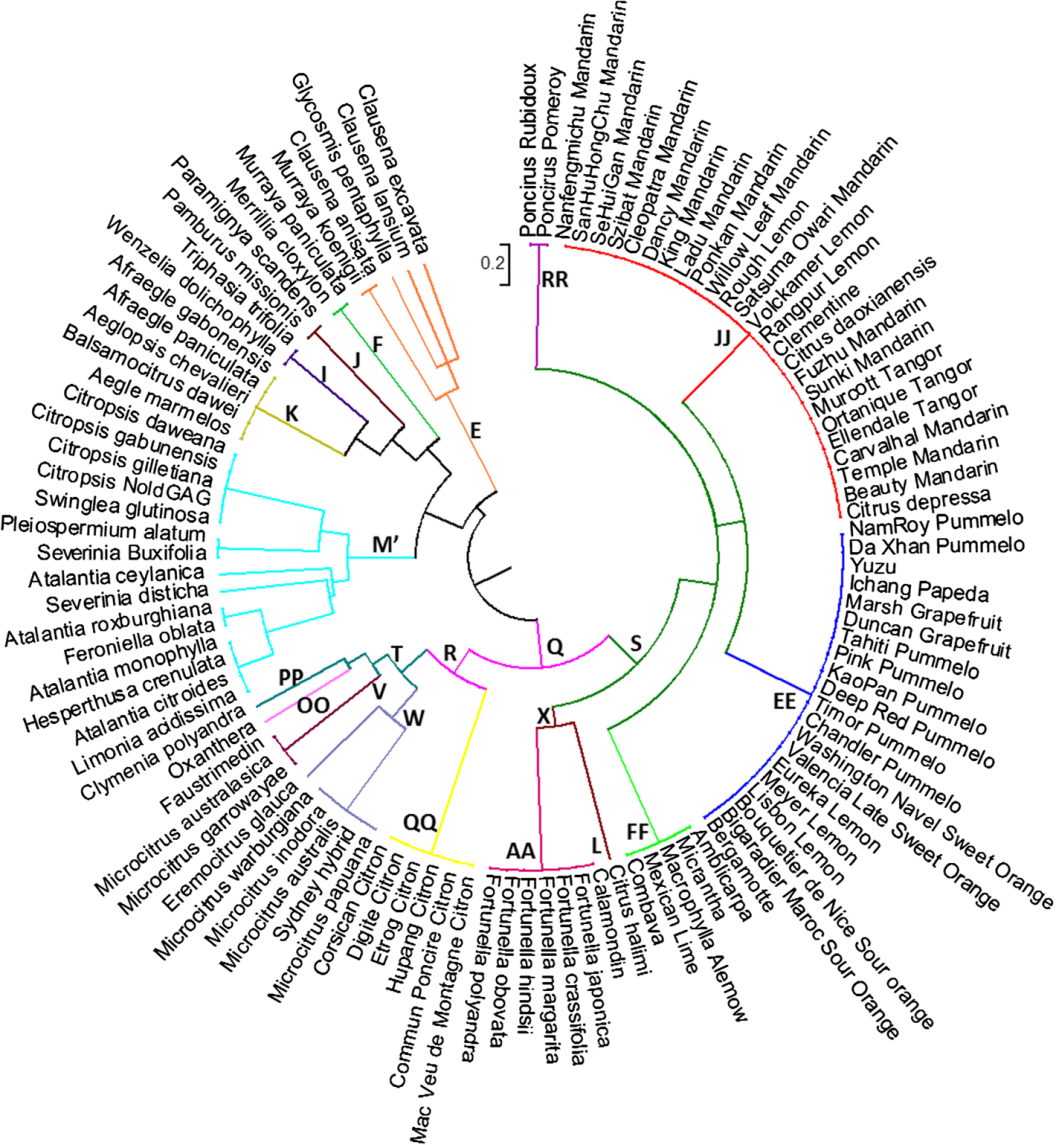
Classification of 108 accessions of the Aurantioideae subfamily using 27 SNP Bayer’s Clade diagnostic markers; the different colors correspond to different clade levels according to the Bayer et al. classification (2009)

An UPGMA analysis was performed with the 27 SNP markers (Fig. 3). The representation was highly coherent with the clade of Bayer et al. [31].

The six genera of the true citrus of Swingle and Reece [13] were included in the Q clade as well as the representative of the genus *Oxanthera* as proposed by Bayer et al. [31]. However, the representative of the genus *Feroniella* (*F. oblata*) was found in the M’ clade, closely associated with representatives of the Balsamocitrinae (as in the Swingle and Reece classification [13]), primitive and near citrus fruits, and is not in the Q clade as concluded by Bayer et al. [31]. Therefore, the genetic conformity of the accession of *Feroniella* used in Bayer et al. [31], or in our study, can be questioned.

Within the Q clade the general organisation globally tallied with 15 of the Bayer clades encountered (with only the position of Yuzu and Ichang papeda in the pummelo clade being different). The Poncirus clade (RR) was clearly differentiated. The mandarin clade (JJ) was largely extended to 14 species of the Tanaka classification (*C. depressa*, *C. tangerina*, *C. daoxianensis*, *C. clementina*, *C. kinokuni*, *C. erythrosa*, *C. suhuiensis*, *C. reticulata*, *C. reshni*, *C. nobilis*, *C. paratangerina*, *C. sunki C. deliciosa*, *C. unshiu*); the supposed natural tangor Ortanique and Murcott also share this clade as concluded by Garcia-Lor et al. [43] from chloroplastic SSRs and mitochondrial indels. Three acid citrus (Rangpur lime, Rough lemon and Volkameriana) were also associated with this clade in agreement with Bayer et al. [31] for Rangpur lime and Rough lemon. For these three acid citrus used as rootstocks, we confirmed the conclusions of Froelicher et al. [18] with mitochondrial indels, and of Curk et al. [45] derived from a large combined cytoplasmic and nuclear analysis demonstrating that these three limes and lemons result from direct hybridization between the mandarin and citron gene pools. With the available data from SNP mining, we were not able to develop markers of the differentiation between sweet and sour mandarin observed with mitochondrial data [18], chloroplastic SSRs [43] and WGS data [10]. The recent availability of these WGS data will certainly make it possible to develop SNP KASPar markers for this differentiation. The pummelo clade (EE) included all the analysed pummelos, sour oranges, sweet oranges, and grapefruits, Meyer and Lisbon lemons, Ichang papeda and Yuzu. The pummelo maternal phylogeny of sour and sweet oranges, and grapefruit was already described by several authors [10, 16–18, 39–41, 53]. Nicolosi et al. [16] proposed that *C. lemon* should be a hybrid between sour orange and citron and it was definitively demonstrated by Curk et al. [45], as well as the pummelo maternal phylogeny of Meyer lemon and Bergamot. The development of two additional SNP markers diagnostic of the differentiation (i) between sour oranges and pummelos, already revealed by chloroplastic SSRs [45] and WGS data [10] and (ii) between sweet oranges pummelo and sour orange revealed by WGS [10] would allow a very fine definition of this clade with few SNP markers. The FF clade (Mexican lime) included also the Combava (*C. hystrix*), *C. micrantha*, *C. macrophylla* and *C. amblycarpa*. These results are in agreement with those of Froelicher et al. [18], Garcia Lor et al. [43] and Curk et al. [45] from mitochondrial indels and chloroplastic SSRs. The six analysed *Fortunella* species were identical (clade AA) and were grouped in clade X with *C. halimii* according to Bayer et al. [31]. These findings confirm the studies of Roose [54], Scora [55], the isozymatic data of Herrero et al. [56], the chemotaxonomy study of Ogawa et al. [57], along with cpRFLP data [58] and nuclear gene data [59]. This genetic relationship should be related to the occurrence of *Fortunella* and *C. halimii* in the Malaysian Peninsula [58]. All citrons were grouped in clade QQ which was joined in clade R with all the Australian and New Caledonian citrus. This relationship between *C. medica* and the Australian/New Caledonian citrus was first revealed by Pfeil et al. [60] and Bayer et al. [31] and recently confirmed by a chloroplastic WGS analysis [10]. Within the T clade of the Australian and New Caledonian citrus, the organisation displayed by Bayer et al. [31] was mostly conserved within clades V and W.

The M’ clade corresponded to the M clade of Bayer et al. [31] but it did not include the Q clade of the true Citrus and *Oxanthera*. This may have been due to the selection of two DSNPs of clade Q. M is the clade where few DSNPs of the Bayer sub-clade were found and where the within genetic organization was not in agreement with Bayer et al. [31] nor with Swingle and Reece [13]. More molecular data would be necessary to have good resolution for this group. Similarly, Penjor et al. [36] and Morton et al. [29] did not support the distinction in the Citrinae sub-tribe between “primitive citrus fruit trees” and “near citrus fruit trees” made by Swingle and Reece [13].

The main clades for the rest of the Citreae and the Clauseneae is respected compared with the Bayer et al. [31] classification with (i) the K clade corresponding to *Afraegle*, *Aeglopsis*, *Balsamocitrus* and *Aegle*; (ii) the I clade joining *Wenzelia* and *Triphasia trifolia*; (iii) the J clade with *Pamburus* and *Paramignya*; (iv) the F clade with *Merrillia* and *Murraya paniculata* and finally (v) the *Clauseneae* clade (E) with *Murraya koenigii*, *Clausena* Sps and *Glycosmis pentaphylla*. An in-depth discussion on the meaning of this cladogram and its relations with previous data can be found in Bayer et al. [31]. To date their work remains the most in-depth at this taxonomic level.

### Usefulness and complementarity of KASPar DSNPs analysis compared with others molecular markers

Although cpDNA analysis for phylogenetic studies is especially based on noncoding regions as well as gene sequence data analysis [37, 38, 41, 52, 61], several studies analysed Aurantioideae maternal phylogeny using different molecular markers such as RFLPs [26, 34, 35, 38, 52, 53, 58], cpSSRs [42–44], Single Strand Conformation Polymorphisms (SSCPs) [62, 63] and CAPS [39, 46]. The complete sequence chloroplast genome of *C. sinensis* [33] led on to in-depth studies such as the use of WGS to elucidate citrus phylogenetic relationships and divergence time estimations [10], and opened up the way for SNP marker development over the whole chloroplast genome. SNPs are the most abundant type of sequence variation in eukaryotic genomes [64, 65] and are considered to be the simplest, the ultimate and the smallest unit of inheritance. Chloroplast SNPs where identified in *Populus* species and a CAPS approach was developed to reveal polymorphisms between seven species [66]. The main disadvantages of the CAPS approach are low throughput and the relatively high cost of genotyping. A large set of soybean chloroplast SNP markers was identified, selected and successfully included in a genotyping array [67], as has been done for potato, *Solanum tuberosum* [68]. However, the use of genotyping arrays remains costly and does not allow flexibility for adaptation to specific research questions needing only a limited specific subset of markers. In our lab the genotyping of one sample for one diagnostic SNP with the KASPar method cost around 0.10 euros per plant sample. For the first time in plant taxonomy, we have shown proof of the concept that a well-selected set of SNPs at subfamily level combined with an efficient competitive allele-specific PCR method (KASPar technology) opens up the way for implementing a highly effective, simple and low-cost molecular taxonomic key.

For recently diverged groups such as the “true citrus” that include the cultivated forms, the recent release of WG chloroplast data for 34 accessions and the corresponding phylogenetic analysis [10] testify that diagnostic polymorphisms of the differentiation between the different genera and all Swingle and Reece *Citrus* species could be found. Therefore, regarding the “true citrus”, for the maternal phylogeny point of view, the set of marker may be complemented and the developed approach will provide a very efficient cladistics molecular key at interspecific level. It is clear, however, that the chloroplast phylogeny reveal only a part of the evolutive history of a gene pool due its mono-parental heredity and should not be enough resolutive for very recently diverged group due to its low rate of variation. Therefore chloroplast and nuclear data are totally complementary to clarify phylogenomic structure and evolutionary stories of recent divergent groups particularly when reticulation events were frequents. It is the case for cultivated *Citrus* arising from hybridisation between four ancestral taxa and displaying nuclear interspecific admixture [20, 22]. Diagnostic nuclear markers of these four taxa coupled with cytoplasmic information were powerful to decipher the origin of citrus secondary species [23, 45] and WGS data provided a detailed picture of the nuclear interspecific mosaic phylogenomic structures of some cultivars [69]. Next Generation Sequencing (NGS) coupled with a reduction of genome complexity using methods such as Genotyping By Sequencing (GBS) or Restriction site Associated DNA Sequencing RAD- seq appears as a good option to provide phylogenomic pictures over the whole nuclear genome complementary to the information given by CpDNA DSNPs.

## Conclusions

CpDNA analysis is a powerful approach for phylogenetic studies on a wide taxonomic level. Monoparental inheritance is particularly useful in a gene pool with reticulate evolution, such as cultivated citrus, to infer the maternal phylogeny. Several methods have been developed for cpDNA analysis, such as CAPS and SSR markers, amplicon sequencing and more recently the use of WGS data mapped in the chloroplast reference genome. In this work, for the first time, we propose using SNPs that are diagnostic of the different clades to establish a molecular taxonomic key of the Aurantioideae. The concept was to focus the molecular analysis on targeted polymorphisms with high taxonomic value and to analyse those polymorphisms with a simple, cost effective, but highly robust genotyping method having wide transferability across the Aurantioideae subfamily. Diagnostic-Clade SNP mining was performed in silico from publicly available cpDNA sequences considering the classification and clades identified by Bayer et al. [21] as the reference. We then tested the efficiency of the competitive allele-specific PCR method (KASPar) to achieve our objectives of wide transferability. From the 40 DSNPs selected from the in silico mining analysis, 27 KASPar markers, diagnostic of 24 Bayer clades, were successfully developed with a very high rate of transferability in the Aurantioideae subfamily (only 1.2 % of missing data on average). Twenty-one key clade markers, univocally diagnostic of 19 clades, were identified. We have demonstrated proof of the concept that with well-established clades, DSNPs can be selected and efficiently transformed into competitive allele-specific PCR markers allowing cost-effective, highly efficient cladistic analysis in large collections at subfamily level. The robustness of the method is a decisive advantage compared with other kinds of cpDNA markers for network collaborative research. The application is considerably easier (laboratory work and data analysis) than a comparison of targeted sequences of amplicons. The availability of WGS data for the main true citrus species may soon enable the development of a set of DSNP markers allowing very fine resolution.

## Additional files

Additional file 1: Sequence accession (GenBank) used for SNP in silico mining and their classification in the Bayer et al. [21] analysis. (XLSX 20 kb) Additional file 2: List of plant material used for KASPar analysis. (XLSX 143 kb) Additional file 3: List of the selected SNPs for KASPar marker development with the sequences of surrounding 50 bases each side and the Bayer et al. (2009) clade theoretically differentiated. (XLSX 13 kb) Additional file 4: Example of diagnostic SNP mining in the region *rps4*-*trnT* in the position 49301 distinguishing the accessions of clade Q (A) from all other Aurantioideae accessions (G). (XLSX 38 kb) Additional file 5: Sequence alignment of all Aurantioideae accessions for the eight chloroplast regions. (XLSX 3440 kb)

## Abbreviations

CAPS: Cleaved amplified polymorphic sequence
cpDNA: ChloroPlastic DNA
DNA: Deoxyribo nucleic acid
DSNP: Diagnostic SNP
FRET: Fluorescent resonance energy transfert
Indel: Insertion/deletion
NCBI: National center of biotechnology information
PCR: Polymerase chain reaction
PIC: Polymorphism information content
RFLP: Restriction fragment length polymorphism
SNP: Single nucleotide polymorphism
SSCP: Single strand conformation polymporphisms
SSR: Simple sequence repeats
UPGMA: Unweighted pair group method with the arithmetic average
WGS: Whole genome sequence
